# Resource niche overlap promotes stability of bacterial community metabolism in experimental microcosms

**DOI:** 10.3389/fmicb.2015.00105

**Published:** 2015-02-24

**Authors:** Ellard R. Hunting, Martina G. Vijver, Harm G. van der Geest, Christian Mulder, Michiel H. S. Kraak, Anton M. Breure, Wim Admiraal

**Affiliations:** ^1^Department of Conservation Biology, Institute of Environmental Sciences (CML), Leiden UniversityLeiden, Netherlands; ^2^Department of Aquatic Ecology and Ecotoxicology, Institute for Biodiversity and Ecosystem Dynamics, University of AmsterdamAmsterdam, Netherlands; ^3^National Institute for Public Health and the Environment (RIVM-LER) - Centre for Sustainability, Environment and HealthBilthoven, Netherlands; ^4^Department of Environmental Science, Institute for Water and Wetland Research, Radboud UniversityNijmegen, Netherlands

**Keywords:** decomposition, functional redundancy, niche complementarity, niche overlap, redox potential, wavelet transform

## Abstract

Decomposition of organic matter is an important ecosystem process governed in part by bacteria. The process of decomposition is expected to benefit from interspecific bacterial interactions such as resource partitioning and facilitation. However, the relative importance of resource niche breadth (metabolic diversity) and resource niche overlap (functional redundancy) on decomposition and the temporal stability of ecosystem processes received little scientific attention. Therefore, this study aims to evaluate the effect of an increase in bacterial community resemblance on both decomposition and the stability of bacterial metabolism in aquatic sediments. To this end, we performed laboratory microcosm experiments in which we examined the influence of bacterial consortia differing in number and composition of species on bacterial activity (Electron Transport System Activity, ETSA), dissolved organic carbon production and wavelet transformed measurements of redox potential (Eh). Single substrate affinities of the individual bacterial species were determined in order to calculate the metabolic diversity of the microbial community. Results presented here indicate that bacterial activity and organic matter decomposition increase with widening of the resource niche breadth, and that metabolic stability increases with increasing overlap in bacterial resource niches, hinting that resource niche overlap can promote the stability of bacterial community metabolism.

## Introduction

Decomposition of organic matter is a central ecosystem process, in which microbial decomposers play a key role by transferring carbon and energy from dead organic matter to higher trophic levels (Odum and de la Cruz, 1963; Gessner et al., 2010). Bacterial decomposers in particular have been viewed as functionally redundant because of a high degree of similarity among bacterial species (e.g., Langenheder et al., 2005; Jiang, 2007; Gamfeldt et al., 2008; Bell et al., 2009 and references therein). Therefore, ecosystem processes like decomposition are supposed to proceed independently from bacterial diversity. However, there is growing evidence indicating that an increase in bacterial diversity positively affects community metabolism (e.g., Bell et al., 2005; Strickland et al., 2009; Wittebolle et al., 2009; Peter et al., 2011).

Bacterial metabolism (e.g., often measured as respiration) has shown to be positively related to bacterial species richness (e.g., Bell et al., 2005, 2009; Peter et al., 2011), while in turn the composition of organic matter has been observed to act as a key driver governing bacteria-mediated processes (e.g., Fonte et al., 2013). This suggests that the processing of organic matter in aquatic sediments is concurrently affected by bacterial diversity and resource diversity. As an alternative to taxonomic diversity (e.g., species richness), it has therefore been argued that metabolic diversity, i.e., the breadth of resource niches of the entire community, provides a better predictor of ecosystem functioning (Hector and Bagchi, 2007; Salles et al., 2009; Peter et al., 2011; Hunting et al., 2013a). This approach has only recently been considered for bacterial communities. The observed positive effects of wider resource niche breadths on denitrification rates and productivity in liquid culture microcosms illustrate its importance for ecosystem processes (Salles et al., 2009; Gravel et al., 2011). However, the relative importance of functional redundancy, e.g., the overlap between resource niches of individual bacteria, for bacterial mediated processes remains uncertain and controversial, which is largely caused by our limited understanding of bacterial functional attributes that are relevant for ecosystem processes (cf. Wohl et al., 2004; Allison and Martiny, 2008).

It is also speculated that bacterial diversity may be equally important for the temporal stability of ecosystem processes (Prosser et al., 2007; Bell et al., 2009; Griffin et al., 2009). However, in contrast to the widely recognized and relatively well studied influence of diversity on the efficiency of ecosystem functioning, the influence of resource niche breadths on the temporal stability of ecosystem processes received little scientific attention and requires further investigation. This is most likely due to the methodological challenge to capture temporal dynamics in bacterial community structure and metabolism. However, increasing evidence suggests that continuous measurements of redox potential (Eh) are valuable for tracking bacterial metabolic activities in time in laboratory microcosms (e.g. Ibarra-Junquera et al., 2006; Rabaey et al., 2007; Escalante-Minakata et al., 2009; Hunting and van der Geest, 2011). Bacterial membrane-bound and extracellular compounds create species specific, redox mediator dependent conditions at the surface of redox electrodes, which are visible as species specific continuous Eh measurements (Brasca et al., 2007; Reichart et al., 2007; Michelon et al., 2010; Tachon et al., 2010; Hunting and Kampfraath, 2013). Mixed bacterial communities generate a composite redox potential and Eh measurements have been shown to reflect relative metabolic activities of members of the community (Peiffer et al., 1992; Ibarra-Junquera et al., 2006; Escalante-Minakata et al., 2009). Continuous measurements of Eh under controlled laboratory conditions could thus provide the unique opportunity to monitor for the first time the effect of increasing bacterial community complexity toward an overlap in resource niches on the temporal changes in bacterial community metabolism in aquatic sediments.

Diverse bacterial communities are likely more efficient in decomposing organic matter as a result of collective resource utilization enabled by resource partitioning or facilitation (e.g., Strickland et al., 2009; Gravel et al., 2011), in which resource complementarity facilitates a stable, continuous utilization of the available substrates. In addition, increasing niche overlap would allow for more stable metabolic processes as this would increase the likelihood of compensatory metabolism by bacterial species with similar resource niches upon fluctuating species abundances (e.g., Yachi and Loreau, 1999). It is therefore hypothesized that wider resource niche breadths promote organic matter decomposition and stability of community metabolism until a high degree of functional redundancy. To begin to test this assumption, this study uses laboratory microcosms to evaluate the influence of bacterial consortia with increasing resource niche overlap on (1) organic matter decomposition and (2) variations in redox potential (Eh) in aquatic sediments.

## Materials and methods

Experiments were performed in microcosms consisting of 50 mL glass vials (Ø 25 mm), ignited quartz sand (12.5 g; grain size: 0.1–0.5 mm) and freeze dried, ground and sieved stinging nettle (*Urtica dioica*, <500 μm, 8 mg) as detrital material. All materials used for the experiments were autoclaved for sterilization and the microcosms were ozonated (800 mg.h^−1^) until the start of the experiment.

Bacterial consortia were assembled from a pool of 12 bacterial strains containing aerobic respirers and denitrifyers that are commonly found in aquatic sediments, including *Azospirillum brasilense, Bacillus subtilis, Paenibacillus polymyxa, Pseudomonas putida, Sphingomonas paucimobilis, Micrococcus luteus, Streptomyces antibiotica, Pseudomonas stutzeri, Flavobacterium* sp., *Aeromonas salmonida, Paracoccus pantotrophus* and *Aminobacter aminovarans* (all obtained from the Netherlands Culture Collection of Bacteria, NCCB, and originally collected in aquatic sediments). Strains were grown in brain-heart broth (MERCK) and peptonised milk nutrient (Sigma) with a ratio of 100:15. Bacterial community were assembled to create a total of 7 richness levels as depicted in Table 1 to obtain an increase in metabolic diversity. This small species pool forced microbial communities to become increasingly similar and functionally redundant with increasing species richness at relatively low levels of diversity. For 5 of these richness levels, three treatments (with *n* = 3 replicates) contained the same number of species, but differed in species composition (Table 1). For the other 2 richness levels, a single treatment (*n* = 3) consisting of the same number of species (11 or 12) and the same species composition was analyzed. To avoid risks of contamination, bacterial strains were not washed prior to assembly and the original growth medium was included in the inoculum. Absorbance was measured at 600 nm in order to standardize optical density at 0.6 of the inoculum, in which each treatment received 1 mL of pre-assembled consortia. In this way we attempted to standardize bacterial biomass at the start of the experiment, although it is possible that based on optical density alone bacterial inoculums differed in bacterial biomass.

**Table 1:** **Design of bacterial consortia assembly, consisting of 7 species richness levels (2–12 species), represented by 3 treatments (*n* = 7) differing in species compositions (A–C)**.

**Number of species**	**Community composition**		
	**A**	**B**	**C**
2	1,2	6,7	11,12
3	1,2,3	6,7,8	10,11,12
4	1,2,3,4	5,6,7,8	9,10,11,12
5	1,2,3,4,5	5,6,7,8,9	8,9,10,11,12
7	1,2,3,4,5,6,7	4,5,6,7,8,9,10	7,8,9,10,11,12
11	1–11		
12	1–12		

*1, Azospirillum brasilense; 2, Bacillus subtilis; 3, Paenibacillus polymyxa; 4, Pseudomonas putida; 5, Sphingomonas paucimobilis; 6, Micrococcus luteus; 7, Streptomyces antibiotic; 8, Pseudomonas stutzeri; 9, Flavobacterium sp.; 10, Aeromonas salmonida; 11, Paracoccus pantotrophus; and 12, Aminobacter aminovarans*.

Bacterial community metabolic diversity (CMD) was pre-determined by evaluating the substrate affinity of each bacterial strain *sensu* Salles et al. (2009) and Gravel et al. (2011) on 96 multiwell plates (Biolog GN®) containing 95 different carbon sources. CMD in the sediment was assessed by community level physiological profiling (CLPP) using Biolog GN microplates containing 95 unique single substrates (Biolog, Inc., Hayward, USA; Garland and Mills, 1991). Biolog GN plates are comprised of simple, common substrates (e.g., sucrose, mallose, and citric acid), and do not include e.g., recalcitrant substrates nor specific substrates typical of the OM used in this study. It is therefore impossible to directly relate substrate utilization profiles to the actual functioning of the developed bacterial communities. Nonetheless, the number of substrates used can serve as a proxy of the metabolic diversity of the bacterial community, and differences in utilization profiles indicate that functionally distinct bacterial communities can develop depending on treatment (Garland, 1999; Hunting et al., 2013b,c). CMD was calculated as the sum of unique substrates used by the assembled bacterial consortium. Overlying water was aerated throughout the experiment with needles, in which we used compressed air sterilized with 0.2 μm Millipore air filters.

Redox potential (Eh) was measured continuously using permanently installed redox microelectrodes and a calomel reference electrode connected to a Hypnos 3 datalogger (Vorenhout et al., 2011) to obtain measurements of 1 mm resolution in the upper (0–7 mm) sediment layer with readings every 15 min. At the end of a 13-day incubation period, organic matter processing was determined by measuring the amount of dissolved organic matter in the overlying water, visible as UV absorbance at 280 nm (Steffen et al., 2002). Previous experiments showed that the absorbance at 280 nm of the substrate used in this study (ground *Urtica dioica*) was positively related to weight loss on ignition (LOI) and accumulation of dissolved inorganic carbon (DIC) in the overlying water (Pearson *r* = 0.853, *p* < 0.001; *r* = 0.896, *p* < 0.0001 respectively) (Hunting et al., 2012). Bacterial activity in the sediment was also determined after 13 days by measuring electron transport system activity (ETSA) following the reduction of 2-(p-iodophenyl)-3-(p-nitrophenyl)-5-phenyl tetrazolium chloride (INT) to formazan (INTF). In brief, 1 mL sediment was collected, vortexed with 1 ml of overlying water and centrifuged (short spin) to deposit course material. Supernatants (400 μL porewater with suspended bacteria) were subsequently assayed for ETS-activity following procedures as recently described (Hunting et al., 2010).

### Data analysis

Wavelet transform spectral analysis is a valuable approach in evaluating spatial and temporal patterns in ecology, and was proven successful in deriving the relative contributions of bacterial species-specific metabolism from continuous measurements of Eh (Bradshaw and Spies, 1992; Ibarra-Junquera et al., 2006; Escalante-Minakata et al., 2009). Temporal Eh measurements in this study were analyzed following a continuous Morlet wavelet transformation *sensu* Bradshaw and Spies (1992). The wavelet transform is a collection of convolutions of the data function, *f*(*x_i_*), (where *x_i_* is a datapoint along a time-series) with a windowing function (or “wavelet”) *g*(*x/a*) for a given range of scales, *a*, centered at locations *x_j_* within the time-series. It is defined as:

$$W(a,x_{j})=\frac{1}{a}\sum_{i=1}^{n}f(x_{i})g\left(\frac{x_{i}-x_{j}}{a}\right)$$

Because the wavelet transform is a function of both scale and location, the interpretation of the resultant transform may be quite difficult for complex patterns. One way to facilitate analysis of temporal variation and comparison between data sets is to calculate the wavelet variance function:

$$V(a)=\frac{1}{n}\sum_{j=1}^{n}W^{2}(a,x_{j})$$

where *n* is defined as the length of the data vector. To create a composite measure of temporal variance for each treatment over time, the wavelet variance is averaged over the combined readings of the individual Eh-probes:

$$V_{t}(a)=\frac{1}{n}\sum_{j=1}^{n}V(a)$$

This approach detects the amplitude of temporal variation of Eh measurements, in which information on the specific timeframes of variability is maintained. These temporal variances, *V_t_*(*a*), were used to obtain the mean overall variance in each treatment, *V_t_*, and temporal stability was subsequently determined as the reciprocal of these measures of variation, 1/*V_t_* (Griffin et al., 2009). Temporal stability of bacterial community metabolism (1/*V_t_*) and organic matter processing were subsequently plotted against species richness (SR) and CMD considering Pearson correlations.

## Results

**Figure 1:**
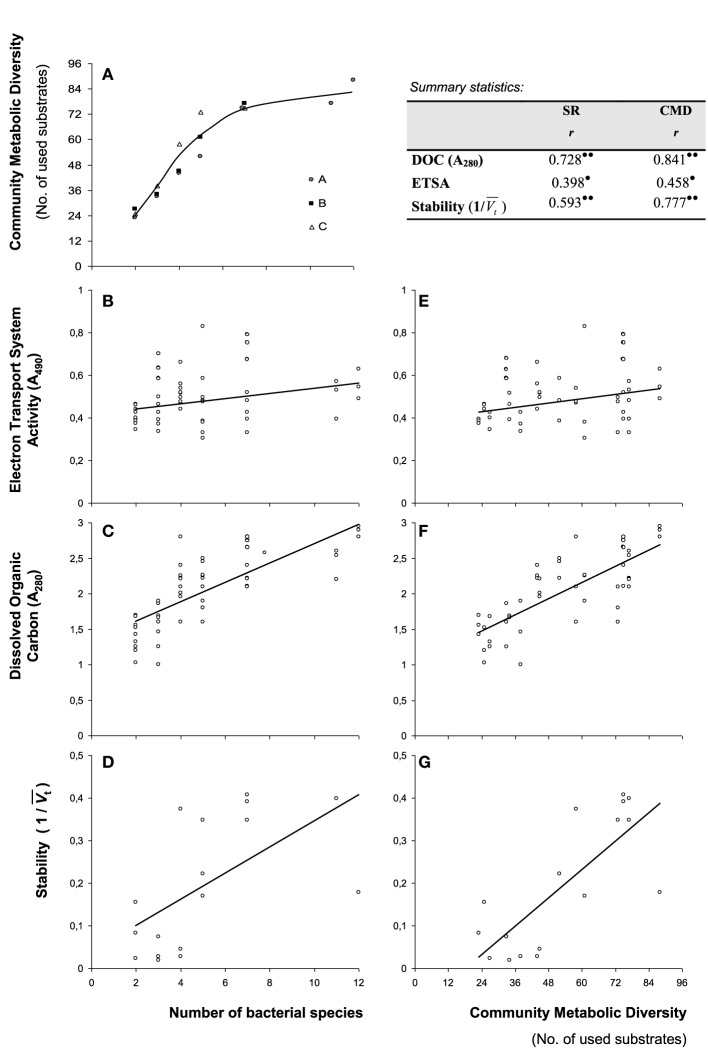
**Correlations of diversity measures and functional parameters**. **(A)** Increase in community metabolic diversity (CMD) with increasing number of bacterial species as determined with single substrate affinities (Biolog GN® plates) of individual bacterial species. Consortia compositions (A–C, following Table 1) are indicated separately and line represents a 3rd order polynomial fit. Correlation of parameters with taxonomic diversity are provided for **(B)** Electron Transport System Activity (ETSA), **(C)** Dissolved Organic Carbon accumulation and **(D)** stability of redox values expressed as the inverse of wavelet variances (1/*V_t_*). Correlations of the same parameters with community metabolic diversity are provided in **(E–G)**. Provided is the summary of correlation statistics of measured parameters depending on species richness (SR) and community metabolic diversity (CMD) based on Pearson correlations (r). Statistical significance indicated as • (*p* < 0.05), and •• (*p* < 0.01).

Bacterial CMD, as pre-determined with substrate utilization affinities of individual bacterial species, increased rapidly with increasing number of species (Figure 1A). CMD and number of species were linearly related up to 5 bacterial species, but reached an asymptote at 7 species (Figure 1A), indicating an increased resource niche overlap.

Bacterial electron transport activity (ETSA) and the amount of dissolved organic carbon liberated upon organic matter degradation after the 13-day incubation period are presented in Figures 1B,C, respectively. There was a slight increase in ETSA with increasing number of species (Figure 1B), in which ETSA and species richness were weakly correlated (Pearson's *r* = 0.398; *p* < 0.05). ETSA was also weakly correlated with CMD (Figure 1E, Pearson's *r* = 0.458; *p* < 0.05). The amount of dissolved organic carbon strongly increased with increasing number of species (Figure 1C), in which DOC and species richness were strongly correlated (Pearson's *r* = 0.728; *p* < 0.01). DOC was also strongly correlated with CMD (Figure 1F, Pearson's *r* = 0.841; *p* < 0.01). Dissolved organic carbon production and ETSA were comparable in bacterial consortia with similar richness levels (Figures 1B,C).

**Figure 2:**
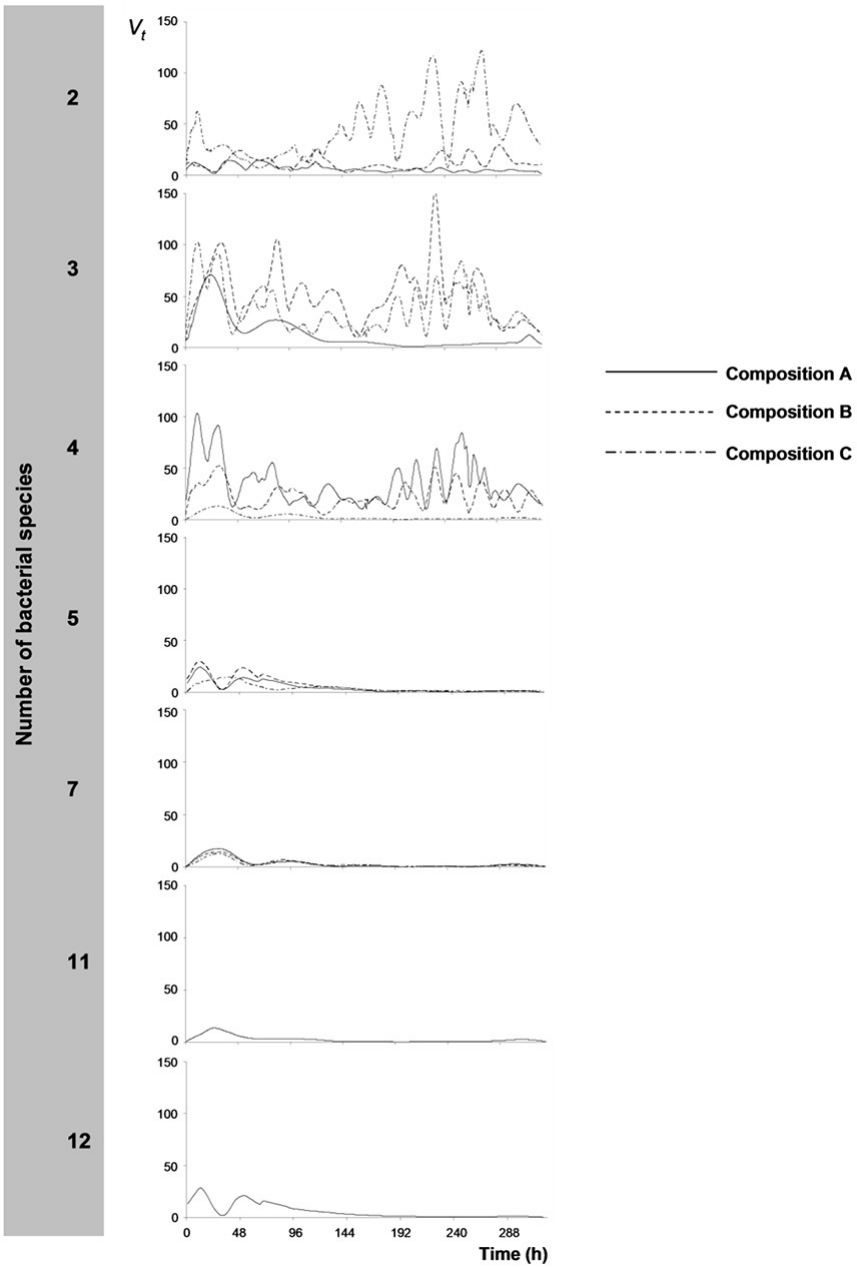
**Wavelet variances, *V*_t_, of redox potential (Eh) time-series**. Wavelet variances are plotted per bacterial species richness level, in which each treatment (species composition, A–C, Table 1) is presented separately.

Wavelet variances, *V_t_*, of continuous measurements of redox potential, Eh, are presented in Figure 2. Wavelet variances were high when bacterial species number and resource niche overlap were low, with variable amplitudes over time, depending on bacterial species composition. In contrast, at increased resource niche overlap (starting at 5 species), *V_t_* decreased and Eh measurements were stable except for some variation at the initial stages of the experiment. Wavelet variances were also expressed as the inverse value, 1/*V_t_*, expressing the metabolic stability of the bacterial consortia. Stability was observed to correlate with SR (Figure 1D, Pearson's *r* = 0.593; *p* < 0.01) and CMD (Figure 1G, Pearson's *r* = 0.777; *p* < 0.01).

## Discussion

Both bacterial activity and organic matter processing increased with an increase in initial number of species and inherent widening of the resource niche breadth of the bacterial community. Although uncertainties exists with respect to the actual succession of bacterial inoculums and we cannot determine the potential influence of competitively dominant and productive species (Cardinale et al., 2006; Jiang, 2007), the observed increased performance in our microcosms appears to illustrate the importance of complementary resource utilization and facilitative interactions among bacterial species on the overall functioning of bacterial communities (Bell et al., 2005; Salles et al., 2009; Peter et al., 2011).

Continuous measurements of redox potential (Eh) became more stable with increasing similarity between the bacterial communities, suggesting that stability of the bacterial metabolic activity coincided with an increase in functional redundancy of the bacterial communities. Stability of continuous redox potential measurements generally corresponds to steady growth of microorganisms and continuous degradation of the available substrate in laboratory microcosms (e.g., Brasca et al., 2007; Reichart et al., 2007; Tachon et al., 2010; Hunting and Kampfraath, 2013). Thus, the observed co-variability in CMD, stability in bacterial metabolism and increased rates of organic matter processing indicates that the metabolic stability of the bacterial community is in part emerging from an efficient, collective resource utilization enabled by resource partitioning. However, increasing stability in bacterial metabolism also coincided with increasing niche overlap. Functional redundancy is expected to increase the likelihood of compensatory metabolism by bacterial species with similar resource niches upon fluctuating conditions (e.g., Yachi and Loreau, 1999), while under stable conditions species are not expected to coexist for long if they rely on the same resources (Gause, 1934). In a comparable experimental setting with stable conditions, Wohl et al. (2004) demonstrated that higher richness levels of functionally redundant (cellulose-degrading) bacterial species can support greater processing rates and facilitate species co-existence. This type of coexistence between functionally redundant species is best explained by differences in the metabolic pathways (Wohl et al., 2004). The major input of organic matter to freshwater sediments is macrophyte or algal derived material (e.g., Wetzel, 1992; Cotner and Biddanda, 2002; Dorgelo et al., 2014) that is mainly polymeric and needs to be hydrolyzed by extracellular enzymes before it can be utilized by microorganisms (e.g., King, 1986; Boschker and Cappenberg, 1998). It is thus possible that all members of the bacterial community mutually benefit from the wider range of enzymes excreted in the sediment matrix, thereby supporting a complementary and stable processing of the available substrates.

Similarly, bacterial species have been observed to occupy an apparent “redox niche” and actively control the redox conditions in their immediate surroundings by membrane bound and secreted redox mediators (Bespalov et al., 1996; Hunting and Kampfraath, 2013). A greater resemblance in the initial bacterial communities increases the likelihood that more bacterial species coexisted and co-operated within the same redox conditions, thereby explaining a stabilized community metabolism and enhanced degradation of organic matter. This mechanism seems supported by earlier findings of co-variability in redox potential, bacterial community structure and metabolism in sediments (Bertics and Ziebis, 2009; Hunting and van der Geest, 2011; Hunting et al., 2012).

Resource composition and availability influence bacterial production and diversity (Judd et al., 2006; Langenheder and Prosser, 2008; Kampfraath et al., 2012; Fonte et al., 2013; Hunting et al., 2013a), while resource history of bacterial communities and complementarity among specialists strongly contribute to productivity and decomposition (Mou et al., 2008; Strickland et al., 2009; Gravel et al., 2011; Langenheder and Székely, 2011). The interplay between resource diversity and bacterial metabolic diversity and community responses to environmental changes thus largely influences the spatio-temporal properties of ecosystem processes. Results presented here indicate that bacterial activity and organic matter decomposition increase with widening of the resource niche breadth, and that metabolic stability increases with increasing overlap in bacterial resource niches. These results were obtained in simplified systems under laboratory conditions and therefore have limited conclusiveness and should be interpreted cautiously. It thus remains uncertain whether patterns observed in the presently studied microcosms reflect those occurring in natural systems. The diversity of natural bacterial communities is very high, and therefore it is commonly thought that natural communities are functionally highly redundant (e.g., Jiang, 2007; Gamfeldt et al., 2008), while ecological filtering by selective environmental pressures typically results in functional redundancy in impaired communities (e.g., Griffiths et al., 2000). Although functional redundancy is typically not expected to impact overall ecosystem processes, our results hint that resource niche overlap can promote stability of bacterial community metabolism.

## Author contributions

EH conceived the study, performed the experiments, analyzed the data and drafted the manuscript. All authors contributed to improve the manuscript and approved the final version of the manuscript.

### Conflict of interest statement

The authors declare that the research was conducted in the absence of any commercial or financial relationships that could be construed as a potential conflict of interest.
